# Knowledge, attitude and practice of HIV/AIDS among employees of a medical line factory in Kamunting, Malaysia

**DOI:** 10.1186/1471-2334-14-S3-P82

**Published:** 2014-05-27

**Authors:** Navinder Kaur, Nik Ahmad Izani, Vivekanandan Gopalakrishnan

**Affiliations:** 1 UNIKL RCMP, 3 Jalan Greentown, Ipoh, Malaysia

## Background

Adults aged between 20 and 39 years constituted for more than half of the new HIV cases in Malaysia. Even though there was a high level of awareness regarding HIV/AIDS among the Malaysian public, there were still misconceptions.

## Methods

It is a cross sectional study conducted at the Latex Manufacturing Sdn.Bhd. Questionnaire was distributed to employees, questions in regard to demographic profiling, knowledge, attitude and practice related to HIV/AIDS were included.

## Results

The study population (n= 314) showed knowledge level with median score of 13/16. However, 46.8% of them could not differentiate between HIV and AIDS, 30.9% believed HIV was transmittable by mosquito bites, 30.6% believed condom usage would not lower HIV risk and 39.5% believed showering or washing genitals after sexual intercourse could lower the risk. Respondents showed good attitude towards people with HIV/AIDS with median attitude score of 5/6. Higher level of education showed higher knowledge level and better (*p*<0.05). About 29% did not use condom during sexual intercourse. Twenty percent had multiple partners and 8% did not use condom regularly with their multiple partners. Twenty seven (8.6%) of male respondents had sexual relationship with other men. About 10% of them were willing to have blood transfusions with blood products of unknown HIV status. High knowledge level did not show better practices related to HIV/AIDS.

## Conclusion

Employees had modest knowledge in regard to HIV/AIDS. Nevertheless, misconceptions and stigmatization in regard to the illness was present, showing that their understanding and concept about the infection was still not satisfactory.

